# The association between urinary metals/metalloids and chronic kidney disease among general adults in Wuhan, China

**DOI:** 10.1038/s41598-023-42282-z

**Published:** 2023-09-15

**Authors:** Yuchai Huang, Zhengce Wan, Mingye Zhang, Liu Hu, Lulu Song, Youjie Wang, Yongman Lv, Le Wang

**Affiliations:** 1 Health Management Center, Tongji Hospital, Tongji Medical College, Huazhong University of Science and Technology, Wuhan, Hubei China; 2 State Key Laboratory of Environment Health (Incubation), Key Laboratory of Environment and Health, Ministry of Education, Key Laboratory of Environment and Health (Wuhan), Ministry of Environmental Protection, School of Public Health, Tongji Medical College, Huazhong University of Science and Technology, Wuhan, China; 3 Department of Maternal and Child Health, School of Public Health, Tongji Medical College, Huazhong University of Science and Technology, Wuhan, Hubei China; 4 Department of Nephrology, Tongji Hospital, Tongji Medical College, Huazhong University of Science and Technology, No.1095 Jiefang Avenue, Wuhan, Hubei China

**Keywords:** Chronic kidney disease, Environmental sciences

## Abstract

The relation between exposure to single metal/metalloid and the risk of chronic kidney disease (CKD) remains unclear. We aimed to determine the single and mixed associations of 21 heavy metals/metalloids exposure and the risk of CKD. We performed a cross-sectional study that recruited 4055 participants. Multivariate logistic regression, linear regression and weighted quantile sum (WQS) regression were conducted to explore the possible effects of single and mixed metals/metalloids exposure on the risk of CKD, the risk of albuminuria and changes in the estimated glomerular filtration rate (eGFR). In single-metal models, Cu, Fe, and Zn were positively associated with increased risks of CKD (*P-trend* < 0.05). Compared to the lowest level, the highest quartiles of Cu (OR = 2.94; 95% CI: 1.70, 5.11; *P*-trend < 0.05), Fe (OR = 2.39; 95% CI: 1.42, 4.02; *P*-trend < 0.05), and Zn (OR = 2.35; 95% CI: 1.31, 4.24; *P*-trend < 0.05) were associated with an increased risk of CKD. After multi-metal adjustment, the association with the risk of CKD remained robust for Cu (*P* < 0.05). Weighted quantile sum regression revealed a positive association between mixed metals/metalloids and the risk of CKD, and the association was largely driven by Cu (43.7%). Specifically, the mixture of urinary metals/metalloids was positively associated with the risk of albuminuria and negatively associated with eGFR.

## Introduction

Chronic kidney disease (CKD) refers to persistent abnormalities in kidney structure or function, and this disease is clinically diagnosed by observing decreased kidney function, markers of kidney damage, or both^1^. The reported prevalence of CKD varies between 10.5% and 13.1% and differs based on race and social class^2–5^. The world health organization (WHO) estimated that the death rate caused by CKD was 12.2 per 100,000 people in 2012 and will increase to 14 per 100,000 people by 2030^6^. Traditionally, CKD was considered to be associated with multiple factors, including genetic sequences, sex, age, lifestyles, environmental factors, and metabolic disorders such as diabetes, hypertension, and cardiovascular diseases^7^. Several recent studies have suggested that metals/metalloids may also harm kidney health and could be related to the incidence of CKD^8,9^.

Metals/metalloids widely exist in daily life and might be chronically ingested by humans via food, drink, air, or skin contact^10,11^. Some metals/metalloids, such as manganese (Mn) and selenium (Se), are essential for health within a certain range of concentrations; other metals/metalloids, including cadmium (Cd), mercury (Hg), and arsenic (As), can be toxic at microconcentrations^12^. Potential mechanisms of toxic effects include the inert form of metals/metalloids, which are determined by their patterns existing in plasma (protein-bound form and ionized form); ion displacement from selected protein sites; and the production of reactive oxygen species^13–15^. However, epidemiological studies that evaluated the associations between metals/metalloids and the risk of CKD have drawn inconsistent conclusions. Researchers found that across different races, levels of metals/metalloids such as lead (Pb), Cd, and As were associated with the risk of CKD^15–17^. However, some other studies have reported nonsignificant associations between these metals/metalloids and CKD^18,19^. An increasing number of epidemiological studies have found that long-term exposure to metals/metalloids might be responsible for decreased renal function and irreversible kidney damage^20^. However, research on the association between metals/metalloids and kidney function has been historically focused on occupational populations under high exposures^21,22^. Few studies have estimated the association between daily metal exposure in healthy and non-healthy subjects. Considering that the human body is simultaneously exposed to multiple metals/metalloids that might have complex interaction effects on physical function in daily life, focusing on the health effects of a single metals/metalloids may not reveal the possible synergistic and antagonistic effects between metals/metalloids and may lead to biased estimates^23,24^. The continuous bioconcentration of metals/metalloids might pose a greater threat to human health^24^. Moreover, most previous studies have examined the influence of single metal or metalloid on CKD^15–19,25^, and few have considered the combined effects of exposure to multiple metals/metalloids.

This cross-sectional study measured 21 kinds of metals and metalloids in the urine of healthy and non-healthy populations from urban central China. We hope to complement existing evidence regarding the effects of daily exposure to individual and multiple metals/metalloids on renal health.

## Methods

### Study population and data collection

We conducted a cross-sectional study to explore the relationships between mixed metals/metalloids and health outcomes in Wuhan, China. From August 2018 to March 2019, 4185 adults were recruited from the health management center of Tongji Hospital, Wuhan, China. Data on demographics, disease history and life style were collected by trained investigators using a pretested questionnaire. Blood and urine samples were generally obtained in the morning after restricting drinking water and overnight fasting. Resting blood pressure and anthropometric data were obtained by trained medical staff following standardized protocol. All participants provided informed consent to be included in the study and have their urine sample tested for metals and metalloids. The ethics committee of Tongji Medical College, Huazhong University of Science and Technology, Wuhan, China approved the study protocol.

The exclusion criteria were as follows: (1) missing value of serum creatine, urinary creatine and urinary albumin (N = 17), and (2) failed to retain urine samples (N = 113).

### Urinary metal detection

Urinary metals/metalloids were detected with the Octagon-based Collision/Reaction Cell (Agilent Technologies) and Agilent 7700X Inductively Coupled Plasma Mass Spectrometer (ICP‒MS).

Morning urine samples of subjects were collected in a microelement-free container and stored at − 20 °C. Samples were pretreated according to the following steps. Then, 500 μL urine was added to 20 μL of 67% (Vol/Vol) HNO_3_ (OptimaTM grade; Fisher Scientific) before being placed into a refrigerator overnight for digestion. Then, 0.5 ml of each sample was mixed and diluted tenfold with 1% (Vol/Vol) HNO_3_. A total of 21 metal concentrations were finally determined: aluminum (Al), zinc (Zn), argentum (Ag), As, barium (Ba), Cd, copper (Cu), iron (Fe), Hg, cobalt (Co), chromium (Cr), cesium (Cs), Mn, Se, strontium (Sr), nickel (Ni), Pb, rubidium (Rb), thallium (Tl), uranium (U), and vanadium (V). Instrument performance was verified each time by applying standard reference materials (SRMs) 1640A (National Institute of Standards and Technology, Gaithersburg, MD, USA) and a blank control (1% HNO_3_). The spiked recoveries of quality control standards ranged from 85 to 120%, and the limits of detection (LODs) varied between 0.00004 μg/L and 0.25 μg/L. Moreover, we used a fully automatic biochemical analyzer (Mindray Medical International Ltd.) to detect the urinary specific gravity (SG) of each sample.

### Renal function assessment

The measurements of serum creatine, urinary creatine, and urinary albumin were carried out by the Department of Laboratory, Tongji Hospital of Tongji Medical College, Huazhong University of Science and Technology. Specifically, serum creatine was detected by the CS-400 series automatic biochemical analyzer (DIRUI CS-800B, Dirui Industrial, China), and urinary microalbumin and creatine were detected by the URIT-500B urine analyzer (URIT Medical Electronic Co., Ltd., China).

The estimated glomerular filtration rate (eGFR) was calculated according to the Modification of diet in renal disease (MRDR) equation based on data from Chinese CKD patients: eGFR = 175 × Scr^−1.234^ × age^−0.179^ [if female, × 0.79]^26^, where Scr is the concentration of serum creatine in mg/dL, age in years. The units for the eGFR were mL/min/1.73 m^2^. According to the kidney disease: improving global outcomes (KDIGO) organization, CKD was defined as either kidney damage or decreased kidney function, or both. Albuminuria, which was defined as an urinary albumin-to-creatinine ratio (ACR) higher than 30 mg/g, was considered an indicator of kidney damage, and decreased kidney function was indicated by an eGFR less than 60 mL/min per 1.73 m^2^^2,7^. Data on the ACR were unavailable for some participants due to the personalized items of physical examination freely chosen by individuals. In our analysis, both the eGFR and ACR were taken into consideration when diagnosing CKD.

### Other variables

After searching the literature and consulting professionals, the following potential confounding factors were assessed in the current study: age (≥ 18 years), gender (male or female), education (lower than high school, high school, or higher than high school), marital status (married or not married), monthly income (≤ 6 000, 6 000–9 999, or ≥ 10 000 RMB), smoking (never, currently or formerly smoking), alcohol (never, currently or formerly drinking), body mass index (BMI), hypertension and diabetes. BMI was calculated by weight in kilograms divided by the square of height in meters. Hypertension was defined as self-reported physician-diagnosed hypertension, intake of antihypertensive medication, or tested blood pressure ≥ 140/90 mmHg. Diabetes was defined as self-reported physician-diagnosed diabetes, intake of antidiabetic medicine, or tested fasting blood glucose ≥ 7.0 mmol/L^27^.

### Statistical analysis

Metals/metalloids concentrations were replaced by limits of detection (LOD) divided by the square root of 2 for those below the LOD^28^. To account for the influence of urine dilution, metals/metalloids concentrations were adjusted for the specific gravity (SG) of urine samples by applying the following equation: C_a_ = C *[SG_median_ − 1]/[SG − 1], where C_a_ represents the SG-adjusted metals/metalloids concentration, C represents the metals/metalloids concentration, and SG_median_ represents the mean SG value of all included samples. The adjusted metals/metalloids concentrations were log-transformed for normal distribution.

Demographic characteristics are displayed as percentages, and the chi-square test was applied for comparisons between participants with or without CKD. The Mann‒Whitney U test was applied to test significant differences of metals/metalloids concentrations between participants with or without data on ACR. Multivariate logistic regression was performed to explore the relationship between a single metal/metalloid and the risk of CKD and albuminuria. Multivariate linear regression was applied to explore the possible effects of a single metal/metalloid on eGFR. Metals/metalloids concentrations were divided into quartiles, and odds ratios (ORs) with their 95% confidence intervals (CIs) were estimated by taking the lowest quartile as a reference. We evaluated both single-metal models and multi-metal models to explore the relationships of every single metal/metalloid with the risk of CKD, albuminuria, and estimated GFR. Single-metal models were adjusted for potential covariates, including age, sex, BMI, marital status, education, family income, smoking, alcohol, hypertension and diabetes. All metals/metalloids that were significant in the single-metal model were subsequently added into multi-metal models, which were adjusted for the same confounders. Moreover, we treated the midpoint concentration of each metal quartile as a continuous variable to test for trends.

Due to the high correlations between metals/metalloids, we used weighted quantile sum (WQS) regression to explore the relationship between the combination of urinary metals/metalloids and the risk of kidney-related outcomes, which is specifically fit for exposures with potential collinearity^29^. Original SG-adjusted concentrations of metals/metalloids were included for constructing WQS regression models. In our analysis, quartile-scored urinary metals/metalloids within the generated mixed index were derived and empirically weighted for each individual based on bootstrap sampling (n = 100), and the weighted quantile score estimated the mean of all estimates. We incorporated the mixed index scores into multivariable regression models adjusted for the previously mentioned confounders. When CKD and albuminuria were the outcomes, multivariable logistic regression models were applied; when eGFR was the outcome, we applied multivariable linear regression models. Both positive and negative models were examined to explore the direction of the relation between mixed metals/metalloids and outcomes. Ultimately, the top six metals/metalloids that accounted for the significant association were displayed along with their weights.

Data analyses were conducted by using R software (version 4.1.0; R Core Team) and SPSS 21.0. A two-sided *P* < 0.05 was considered statistically significant.

## Results

### Characteristics of the study population

A total of 4055 participants were ultimately included in our analysis. Data on eGFR and urine metals/metalloids were available for all the participants, while the ACR was available for 1817 patients and missing for 2238 patients. As a result, 1817 participants were included in the analysis for the effects of metals/metalloids exposure on the risk of CKD and albuminuria, and data from all 4055 participants were analyzed to examine eGFR. A total of 31 participants had decreased kidney function, 129 participants had albuminuria, and 138 participants had CKD.

The demographic characteristics of all participants are shown in Table 1. Among all included participants, 63.60% were males and 9.40% were elderly people. The number of participants in the normal range of BMI was less than half (45.70%). Nearly half of the participants earned more than ten thousand yuan each month (48.60%). More than half of the participants were married (90.10%), had an education higher than high school (64.30%), never smoked (68.60%), or drank alcohol (69.60%). Most participants had no hypertension (73.10%) or diabetes (94.20%). Except for sex and smoking habits (*P* > 0.05), all considered characteristics significantly differed between subjects with and without CKD (*P* < 0.05).

**Table 1:** Distribution of demographic characteristics data are n (%), unless stated otherwise.

Variables N (%)	eGFR as outcome (N = 4055)	CKD as outcome (n = 1817)		
		Yes (n = 138)	No (n = 1679)	*P*
Gender				
Male	2581 (63.60%)	94 (68.10%)	1056 (62.90%)	0.22
Female	1474 (36.40%)	44 (31.90%)	623 (37.10%)	
Age (y)				
18–44	1860 (45.90%)	39 (28.30%)	838 (49.90%)	< 0.05
45–59	1814 (44.70%)	75 (54.30%)	744 (44.30%)	
> = 60	381 (9.40%)	24 (17.40%)	97 (5.80%)	
BMI (Kg/m^2^)				
< 18.5	108 (2.70%)	2 (1.40%)	41 (2.40%)	< 0.05
18.5–23.9	1848 (45.70%)	41 (29.70%)	754 (44.90%)	
24–27.9	1582 (39.10%)	59 (42.80%)	658 (39.20%)	
≥ 28	507 (12.50%)	36 (26.10%)	226 (13.50%)	
Marital status				
Not married	400 (9.90%)	4 (2.90%)	160 (9.60%)	0.01
Married	3630 (90.10%)	134 (97.10%)	1513 (90.40%)	
Education				
Lower than high school	755 (18.90%)	55 (39.90%)	421 (25.30%)	< 0.05
High school	673 (16.80%)	20 (14.50%)	342 (20.60%)	
Higher than high school	2570 (64.30%)	63 (45.70%)	900 (54.10%)	
Monthly income (yuan)				
Lower than 6000	992 (25.90%)	43 (32.80%)	364 (23.10%)	0.04
6000–9999	979 (25.50%)	25 (19.10%)	373 (23.70%)	
High than 10,000	1865 (48.60%)	63 (48.10%)	837 (53.20%)	
Smoking				
Never	2776 (68.60%)	92 (66.70%)	1153 (68.80%)	0.29
Currently smoking	1009 (24.90%)	42 (30.40%)	432 (25.90%)	
Formerly smoking	261 (6.50%)	4 (2.90%)	88 (5.30%)	
Alcohol				
Never	2811 (69.60%)	83 (61.00%)	1142 (68.30%)	< 0.05
Currently drinking	1096 (27.10%)	43 (31.60%)	486 (29.00%)	
Formerly drinking	133 (3.30%)	10 (7.40%)	45 (2.70%)	
Hypertension				
Yes	1090 (26.90%)	88 (62.90%)	418 (24.90%)	< 0.05
No	2965 (73.10%)	52 (37.10%)	1259 (75.10%)	
Diabetes				
Yes	236 (5.80%)	28 (20.00%)	78 (4.70%)	< 0.05
No	3819 (94.20%)	112 (80.00%)	1599 (95.30%)	

Table 2 displays the distribution for each metal and metalloid, including the LODs and percentages lower than the LODs. All subjects had been tested for 21 metals/metalloids. Except for the concentrations of U (*P* > 0.05), participants with missing ACR data seemed to have higher Ag and Hg levels but lower levels of the other 18 kinds of metals/metalloids than subjects with ACR data (*P* < 0.05). Spearman correlation analyses showed that all the metals/metalloids were positively correlated with each other except for Ag (Table S1).

**Table 2:** Distribution of urinary SG-adjusted metals/metalloids concentrations of the study population P_50_(P_25_, P_75_); Based on Mann–Whitney U test.

Metals/Metalloids (ug/L)	LOD	< LOD (%)	Total	With ACR	Without ACR	*P*
Al	0.25	16 (0.39%)	26.96 (16.39, 44.88)	38.27 (23.69, 64.90)	20.89 (13.42, 31.28)	< 0.001
V	0.0005	1 (0.02%)	0.53 (0.37, 0.74)	0.58 (0.41, 0.86)	0.49 (0.35, 0.67)	< 0.001
Cr	0.0036	6 (0.15%)	1.34 (0.71, 2.58)	2.18 (1.29, 3.74)	0.89 (0.51, 1.59)	< 0.001
Mn	0.0023	3 (0.07%)	1.12 (0.65, 2.01)	1.52 (0.93, 2.60)	0.89 (0.53, 1.51)	< 0.001
Fe	0.026	0	21.17 (12.86, 37.88)	30.57 (18, 62, 51.49)	16.01 (9.96, 26.83)	< 0.001
Co	0.0005	5 (0.12%)	0.25 (0.16, 0.44)	0.32 (0.19, 0.56)	0.21 (0.14, 0.34)	< 0.001
Ni	0.013	22 (0.54%)	3.60 (2.02, 7.12)	5.71 (2.91, 11.01)	2.71 (1.65, 4.61)	< 0.001
Cu	0.016	28 (0.69%)	10.73 (7.14, 16.48)	13.33 (8, 49, 21.65)	9.23 (6.21, 13.39)	< 0.001
Zn	0.17	6 (0.15%)	291.91 (173.19, 493.79)	338.02 (202.20, 595.38)	261.10 (157.65, 429.14)	< 0.001
As	0.007	3 (0.07%)	20.99 (12.26, 35.72)	22.45 (12.84, 39.98)	19.88 (11.92, 33.28)	< 0.001
Se	0.015	2 (0.05%)	17.21 (10.99, 27.26)	18.43 (11.42, 29.31)	16.53 (10.64, 25.36)	< 0.001
Rb	0.0007	0	1592.37 (1022.98, 2456.82)	1930.58 (1209.70, 3005.51)	1394.43 (929.08, 2077.97)	< 0.001
Sr	0.0036	0	84.75 (51.10, 135.20)	97.45 (57.98, 156.64)	76.11 (47.25, 118.13)	< 0.001
Ag	0.038	184 (4.54%)	0.21 (0.11, 0.44)	0.13 (0.07, 0.22)	0.36 (0.18, 0.64)	< 0.001
Cd	0.0006	3 (0.07%)	0.62 (0.34, 1.15)	0.68 (0.38, 1.27)	0.58 (0.32, 1.06)	< 0.001
Cs	0.0004	2 (0.05%)	6.28 (4.21, 9.33)	7.37 (4.83, 10.96)	5.65 (3.86, 8.16)	< 0.001
Ba	0.024	54 (1.33%)	3.57 (92.03, 5.99)	4.03 (2.41, 6.43)	3.21 (1.78, 5.56)	< 0.001
Hg	0.00004	16 (0.39%)	0.82 (0.44, 1.46)	0.76 (0.39, 1.46)	0.87 (0, 48, 1.47)	0.004
Tl	0.0002	0	0.31 (0.21, 0.48)	0.39 (0.25, 0.61)	0.27 (0.18, 0.39)	< 0.001
Pb	0.0026	13 (0.32%)	1.66 (1.01, 2.81)	2.21 (1.39, 3.57)	1.34 (0.83, 1.12)	< 0.001
U	0.0002	15 (0.37%)	0.23 (0.01, 0.04)	0.023 (0.014, 0.036)	0.024 (0.014, 0.044)	0.47

## Association between single metal/metalloid and outcomes

### Association of single metal/metalloid with the risks of CKD and albuminuria

**Figure 1:**
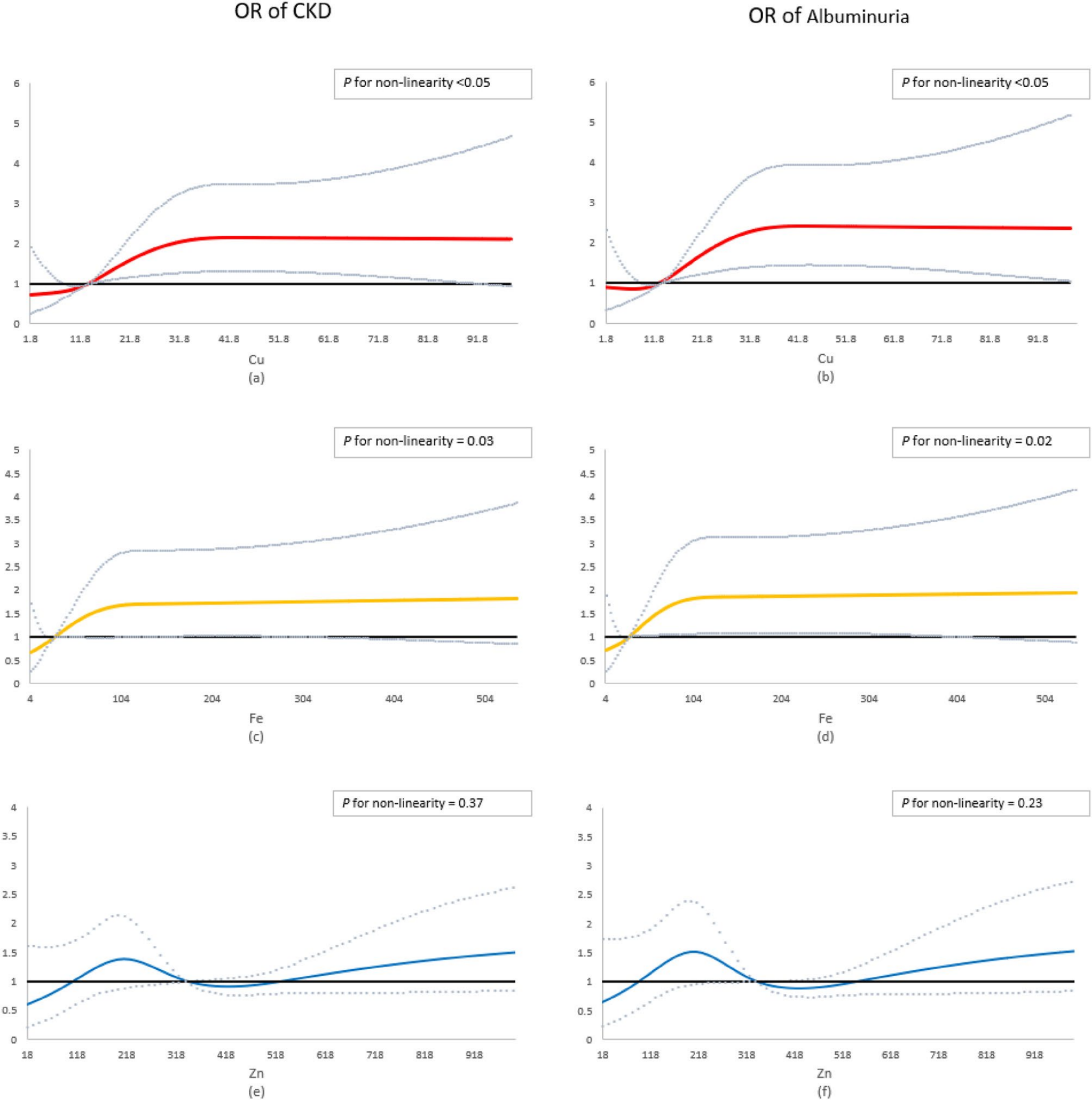
Restrict cubic spline models of the associations between concentrations of urinary metals and risk of CKD and albuminuria. The nonlinear associations for the risk of CKD and albuminuria were significant with Cu (**a** and **b**) and Fe (**c** and **d**), and were non-significant with Zn (**e** and **f**).

Three metals/metalloids were found to be significantly associated with the risks of CKD and albuminuria (Table 3). In single-metal models, Cu, Fe, and Zn were significantly positively associated with increased risks of CKD and albuminuria (*P-trend* < 0.05). Compared to the lowest level, the highest quartiles of Cu (OR = 2.94; 95% CI: 1.70, 5.11; *P*-trend < 0.05), Fe (OR = 2.39; 95% CI: 1.42, 4.02; *P*-trend < 0.05), and Zn (OR = 2.35; 95% CI: 1.31, 4.24; *P*-trend < 0.05) were associated with an increased risk of CKD. For the risk of albuminuria, the effects of Cu, Fe, and Zn at the highest levels were 3.03 (95%CI: 1.72, 5.33), 2.52 (95%CI: 1.48, 4.31), and 2.07 (95%CI: 1.15, 3.75) when compared with the lowest levels respectively. The restricted cubic spline analysis showed significant nonlinear associations of Cu (*P* for non-linear both < 0.05) and Fe (*P* for non-linear both < 0.05) with CKD and albuminuria, while Zn (*P* for non-linear both > 0.05) showed a nonsignificant nonlinear association with CKD and albuminuria (Fig. 1). The other 18 metals/metalloids showed nonsignificant associations with the risks of CKD and albuminuria (Tables S2 and S3). Moreover, the positive effect of Cu on the risk of CKD and albuminuria remained robust in the multi-metal model (*P* < 0.05).

**Table 3:** Associations of single urinary metals/metalloids with the risks of CKD and albuminuria.

Outcome	Metals (μg/L)	Models	Odds ratio (95% CI) by quartile of metals/metalloids				*P-trend*
			Q1	Q2	Q3	Q4	
CKD	Cu	Model 1	Ref	2.35 (1.33, 4.16)	2.11 (1.16, 3.85)	2.94 (1.70, 5.11)	< 0.001
		Model 2	Ref	1.98 (1.07, 3.69)	1.68 (0.83, 3, 42)	2.23 (1.01, 4.91)	0.012
	Fe	Model 1	Ref	2.15 (1.25, 3.68)	1.38 (0.75, 2.56)	2.39 (1.42, 4, 02)	0.005
		Model 2	Ref	1.69 (0.96, 2.98)	0.93 (0.47, 1.82)	1.41 (0.71, 2.82)	0.729
	Zn	Model 1	Ref	1.69 (0.91, 3.13)	2.31 (1.28, 4.18)	2.35 (1.31, 4.24)	0.003
		Model 2	Ref	1.34 (0.70, 2.55)	1.55 (0.80, 2.99)	1.30 (0.63, 2.68)	0.352
Albuminuria	Cu	Model 1	Ref	2.23 (1.23, 4.04)	2.20 (1.19, 4.07)	3.03 (1.72, 5.33)	< 0.001
		Model 2	Ref	1.94 (1.02, 3.71)	1.86 (0.90, 3.83)	2.48 (1.11, 5.55)	0.045
	Fe	Model 1	Ref	2.12 (1.21, 3.73)	1.45 (0.77, 2.74)	2.52 (1.48, 4.31)	0.003
		Model 2	Ref	1.67 (0.93, 3.03)	0.97 (0.48, 1.96)	1.52 (0.75, 3.07)	0.565
	Zn	Model 1	Ref	1.56 (0.84, 2.90)	2.14 (1.18, 3.88)	2.07 (1.15, 3.75)	0.010
		Model 2	Ref	1.20 (0.63, 2.29)	1.34 (0.69, 2.61)	1.01 (0.49, 2.11)	0.816

Model 1 was the single-metal model adjusted for age, gender, BMI, marital status, education, family income, smoking, alcohol, hypertension, diabetes.

Model 2 was the multiple-metal model adjusted for covariates in model1 plus metals/metalloids significant in single-model.

### Association between a single metal/metalloid and eGFR

The significant effects of single metal/metalloid on eGFR are shown in Table 4. In single-metal models, Cu, Zn, Mn, Ni, As, Se, Cd, Cs, and U were significantly associated with reduced eGFR, while Sr was associated with elevated eGFR. Compared to the lowest quantiles, the highest quantiles of Cu, Zn, Mn, Ni, As, Se, Cd, Cs, and U were associated with an eGFR reduction of 2.65 (95%CI: 0.96, 4.33), 3.33 (95%CI: 1.58, 5.09), 2.28 (95%CI: 0.58, 3.97), 2.29 (95%CI: 0.60, 3.98), 2.73 (95%CI: 1.03, 4.42), 2.58 (95%CI: 0.87, 4.28), 2.85 (95%CI: 1.09, 4.61), 1.86 (95%CI: 0.16, 3.56), and 2.11 (95%CI: 0.42, 3.80), respectively. The highest quantile of Sr was associated with 2.58 (95%CI: 0.87, 4.28) of elevated eGFR when compared with the lowest quantile. The other unmentioned metals/metalloids were found to be non-significantly related to eGFR, with a *P-trend* > 0.05 (Table S5). Furthermore, multi-metal models showed that Sr (*P-trend* < 0.05) was still associated with increased eGFR, and As remained significantly related to decreased eGFR (*P-trend* < 0.05).

**Table 4:** Associations between single urinary metals/metalloids and eGFR.

Metals/metalloids	Relative change (95% CI) of eGFR by quartile of metals/metalloids				*P − trend*
	Q1	Q2	Q3	Q4	
Single − metal model					
Cu	Ref	− 0.52 (− 2.20, 1.16)	− 1.60 (− 3.29, 0.09)	− 2.65 (− 4.33, − 0.96)	< 0.05
Zn	Ref	− 1.82 (− 3.52, − 0.12)	− 1.42 (− 3.14, 0.30)	− 3.33 (− 5.09, − 1.58)	< 0.05
Mn	Ref	− 0.48 (− 2.17, 1.21)	− 1.50 (− 3.20, 0.20)	− 2.28 (− 3.97, − 0.58)	< 0.05
Ni	Ref	− 0.41 (− 2.10, 1.28)	− 0.53 (− 2.22, 1.16)	− 2.29 (− 3.98, − 0.60)	0.01
As	Ref	− 1.27 (− 2.96, 0.42)	− 2.63 (− 4.32, − 0.93)	− 2.73 (− 4.42, − 1.03)	< 0.05
Se	Ref	− 0.77 (− 2.47, 0.93)	− 1.82 (− 3.51, − 0.12)	− 2.58 (− 4.28, − 0.87)	< 0.05
Sr	Ref	1.01 (− 0.68, 2.69)	1.86 (0.17, 3.55)	2.09 (0.39, 3.78)	< 0.05
Cd	Ref	− 1.69 (− 3.38, − 0.01)	− 1.43 (− 3.14, 0.29)	− 2.85 (− 4.61, − 1.09)	< 0.05
Cs	Ref	− 1.26 (− 2.95, 0.44)	− 1.77 (− 3.47, − 0.08)	− 1.86 (− 3.56, − 0.16)	0.03
U	Ref	− 1.18 (− 2.87, 0.52)	− 1.74 (− 3.43, − 0.42)	− 2.11 (− 3.80, − 0.42)	0.01
Multiple − metal model					
Cu	Ref	0.10 (− 1.70, 1.90)	− 0.56 (− 2.52, 1.41)	− 0.71 (− 3.03, 1.60)	0.48
Zn	Ref	− 1.46 (− 3.31, 0.38)	− 0.83 (− 2.89, 1.22)	− 2.23 (− 4.63, 0.18)	0.12
Mn	Ref	− 0.28 (− 2.01, 1.45)	− 1.01 (− 2.84, 0.83)	− 1.21 (− 3.24, 0.82)	0.17
Ni	Ref	0.18 (− 1.61, 1.96)	0.60 (− 1.31, 2.52)	− 0.77 (− 2.86, 1.32)	0.62
As	Ref	− 1.45 (− 3.32, 0.42)	− 2.94 (− 5.01, − 0.87)	− 2.97 (− 5.24, − 0.70)	0.01*
Se	Ref	− 0.29 (− 2.27, 1.69)	− 0.90 (− 3.20, 1.40)	− 1.53 (− 4.25, 1.18)	0.24
Sr	Ref	2.51 (0.76, 4.26)	4.04 (2.56, 6.25)	6.11 (4.09, 8.13)	< 0.05*
Cd	Ref	− 1.32 (− 3.10, 0.47)	− 1.02 (− 2.96, 0.93)	− 1.92 (− 4.11, 0.27)	0.12
Cs	Ref	− 0.25 (− 2.26, 1.76)	0.34 (− 1.96, 2.63)	1.67 (− 0.96, 4.30)	0.20
U	Ref	− 0.67 (− 2.42, 1.08)	− 1.01 (− 2.81, 0.80)	− 1.40 (− 3.24, 0.44)	0.13

Single-metal models adjusted for age, gender, BMI, marital status, education, family income, smoking, and alcohol, hypertension, diabetes.

Multiple-metal models adjusted for co-variates in single-metal models plus metals/metalloids significant in single-metal models.

*Represented significant association in multiple-metal model.

## Association between mixed metals/metalloids and outcomes

**Figure 2:**
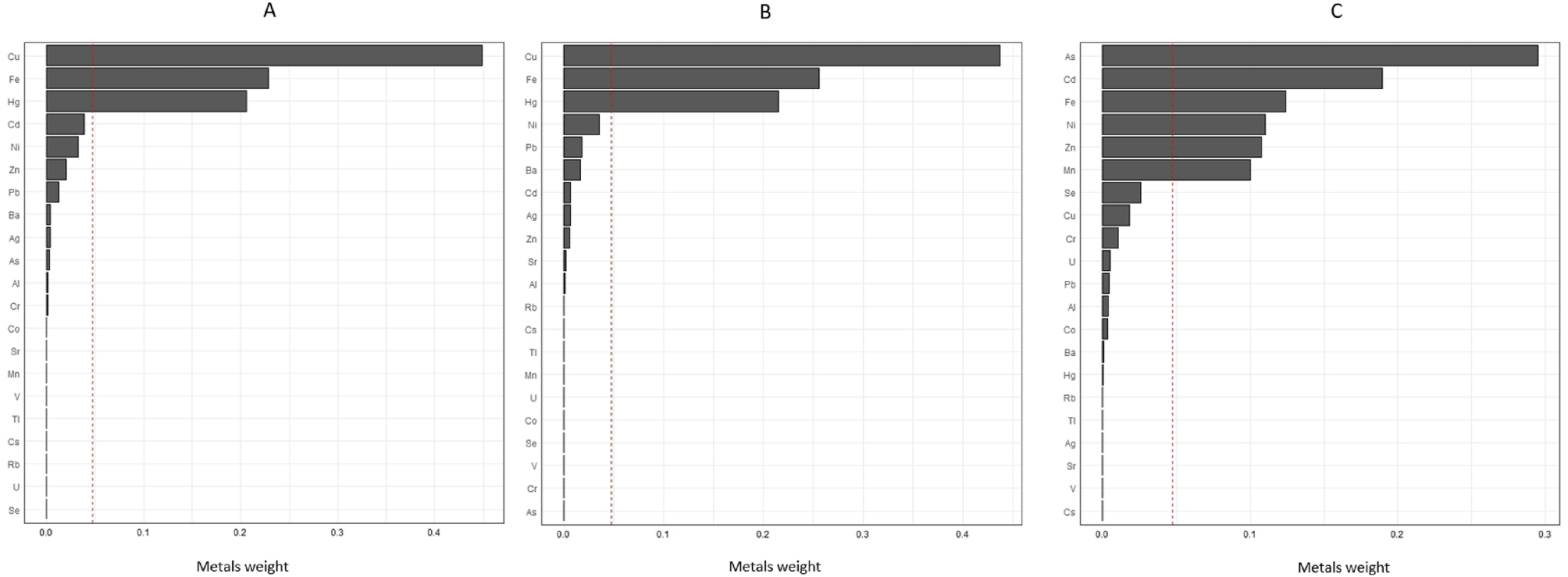
The weights of metals in urine mixture based on weighted quantile sum (WQS) regression. The effect on the risk of CKD (**A**) and albuminuria (**B**) was in a positive direction, while eGFR (**C**) was in a negative direction. Models were adjusted for age, gender, BMI, income, marital status, education, smoking, alcohol, hypertension, and diabetes.

As shown in Table 5, there was a significant positive relationship between urinary mixed metals/metalloids and the risks of CKD and albuminuria, and there was a significant negative relationship between urinary mixed metals/metalloids and eGFR. The increased risk of CKD was primarily attributed to Cu (44.91%), followed by Fe (22.88%) and Hg (20.60%). Similarly, the increased risk of albuminuria was primarily attributed to Cu (43.71%), followed by Fe (25.60%), and Hg (21.46%). The reduction in eGFR was primarily attributed to As (29.50%), followed by Cd (18.96%) and Fe (12.43%) (Table 6 and Fig. 2).

**Table 5:** Associations between mixed metals/metalloids and outcomes.

Outcomes	Estimated WQS index	SE	*P*
Positive direction			
CKD	0.12	0.06	0.02*
Albuminuria	0.19	0.06	0.02*
eGFR	0.28	0.21	0.17
Negative direction			
CKD	− 0.02	0.06	0.79
Albuminuria	− 0.01	0.06	0.98
eGFR	− 0.43	0.20	0.03*

*Represented significant association in WQS regression model.

Models were adjusted for age, gender, BMI, income, marital status, education, smoking, alcohol, hypertension, diabetes.

WQS model was based on linear regression.

**Table 6:** Metals/metalloids and their weights in mixture related to the risk of CKD, albuminuria, and eGFR reduction.

Outcome	Metals/metalloids	Weight (%)
CKD	**Cu**	44.91*
	Fe	22.88
	Hg	20.60
	Cd	3.90
	Ni	3.22
	Zn	2.02
Albuminuria	**Cu**	43.71*
	Fe	25.60
	Hg	21.46
	Ni	3.54
	Pb	1.77
	Ba	1.62
eGFR	**As**	29.51*
	Cd	18.96
	Fe	12.43
	Ni	11.02
	Zn	10.77
	Mn	10.03

*Represented significant association in WQS regression model.

## Discussion

To the best of our knowledge, this was the first study that measured 21 kinds of metals/metalloids simultaneously and evaluated their individual and pooled association with CKD and its components. After performing single-metal, multiple-metal, and mixed analyses, we identified several interesting findings. (1) The risks of CKD and albuminuria were positively associated with urinary Cu, Fe, and Zn concentrations, and the relation with Cu remained robust in multiple-metal models. (2) Estimated glomerular filtration rate was positively associated with urinary Sr concentrations but negatively associated with urinary Cu, Zn, Mn, Ni, As, Se, Cd, Cs, and U concentrations, and the relationship of eGFR with Sr and As remained significant in multi-metal models. (3) The mixture of 21 metals/metalloids was positively associated with the risks of CKD and albuminuria but negatively associated with eGFR; these associations were largely driven by Cu and As, respectively.

Urine is a noninvasive and commonly preferred medium for biomonitoring, especially for water-soluble chemicals, such as metals and metalloids^30^. As urine is produced, the kidney is constantly exposed to many toxins and pollutants, which makes it susceptible to adverse effects. We considered metals and metalloids that may increase the risk of incident CKD or albuminuria or decrease the eGFR to be kidney-unfriendly metals/metalloids (KUMs) or kidney-friendly metals/metalloids (KFMs).

Copper and iron were possible KUMs. In our study, increased Cu and Fe might be related to the increased risk of CKD and albuminuria, as well as accounting for the decrease in eGFR. Both Cu and Fe are important biological oxidation factors. As possible compounds of superoxide dismutase (SOD), Cu and Fe can help resist oxidative damage in cells^31^. However, they are also oxygen reactive factors, which could be dangerous for tubular epithelial cells and cause oxidative cellular damage when overloaded^32,33^. Previous epidemiological and mechanical studies have proposed that high exposure to Cu, whether in serum or urine, may threaten the risks of CKD and albuminuria and cause adverse renal performance, including a decrease in eGFR^9,33–35^. Similarly, for high exposure to Fe, increased urinary iron excretion, as well as damaged renal function, were observed in the experimental study^36^. Furthermore, both experimental and epidemiological studies have reported increased urine iron concentrations in subjects with injured renal function^37,38^. Consequently, the relationship between urine iron and renal function might be bidirectional. The increased exposure to Fe might threaten renal health, which in turn causes the elevation of urine Fe concentration. Further studies are needed for verification.

Zinc is essential for the human body and is involved in many catalytic, structural, and regulatory activities, including the replication of DNA, energy metabolism, maintenance of protein structure, body growth and development^39,40^. Multiple epidemiological studies have found that Zn deficiency may have harmful impacts on human health, such as inducing CKD^33,34^. However, in our study, the highest exposure to Zn was associated with an increased risk of hazardous renal performance, which provides potential evidence for the U-shaped association between Zn and renal health. As a traditional industrial city, Wuhan residents may be exposed to relatively higher metal concentrations than other city dwellers. In our study, the median SG-adjusted urine Zn concentration was 292 µg/L for all included participants, which was higher than studies conducted in the German population (median = 207 µg/L) and close to the median reported in another study conducted in Wuhan (median = 277 µg/L)^41^. A cohort study based on Chinese middle-aged and elderly individuals found that excessive exposure to Zn was positively associated with a rapid decrease in eGFR as well as a decline in kidney function^40^, which is consistent with our results. A possible explanation might be that excessive exposure to Zn might activate kidney angiotensin II and reduce renal blood flow, causing a reduction in renal function^42^. However, such an explanation is still limited to animal experiments. More human research is needed to confirm this hypothesis. As a result, elements friendly for health might also be harmful when they are excessive, and Zn may not be an exception.

We found that metals/metalloids, including Mn, Ni, As, Se, Cd, Cs, and U, might be harmful to kidney function, as they were related to a decrease in eGFR. Cadmium has a half-life as long as 10 to 30 years and can be stored in the liver for a long time before being slowly released into the blood, constantly deteriorating the reabsorption function of renal tubules and reducing kidney function^43,44^. Limited evidence from epidemiological studies suggests that Cd may contribute to poor kidney performance^21,45,46^. In our analysis, the above mentioned 7 metals/metalloids, including Cd, were related to decreased eGFR, and a nonsignificant association was found between these metals/metalloids and the risks of proteinuria or CKD. As evidence from epidemiological studies based on the Chinese population is still limited, further prospective investigations are needed to verify and distinguish the association between metals/metalloids and forms of poor kidney performance. For the general population, arsenic exposure could be mainly attributed to drinking water, which could be rapidly absorbed by the intestine. Arsenic was reported to lead to worse kidney health by blocking sodium and glucose transportation of tubules, causing cortical necrosis and interfering with oxidative phosphorylation in renal tubes^8^. Prospective studies have found that As exposure in plasma and urine are both positively associated with decreased kidney function^40,47^. A recent systematic review and meta-analysis concluded that Cd and As exposure were both associated with an elevated risk of CKD; specifically, arsenic was found to be associated with a greater risk of decreased eGFR, and Cd was related to an increased risk of proteinuria^8^. However, previous evidence of the associations of Mn, Ni, Se, Cs, and U exposure with kidney health is still inconsistent and needs further investigation.

Uranium could be mostly excreted with urine in several weeks, and U toxicity can primarily occur in the kidney^48^. A cross-sectional study found that U exposure might be responsible for the decline in eGFR, even in Chinese hypertension patients^49^. Another study analyzed data from Americans and reported an association between urinary U exposure and incident albuminuria, while no association was found with the decrease in eGFR^50^. A nonsignificant association between U exposure and kidney health performance has also been reported^51^. Further molecular mechanism research and prospective population studies are needed to clarify this issue. A prospective study reported that a high concentration of Ni was a risk factor for the progression of end-stage renal disease (ESRD)^35^. Another longitudinal study based on the Chinese population reported a nonsignificant positive association between plasma Ni, Mn, Se and impaired kidney function measured by decreased eGFR^40^, which was opposite to our finding. Se deficiency has been reported in CKD patients of unknown aetiology^52^. However, to the best of our knowledge, few epidemiological studies have reported an association between Mn, Se, and Cs and renal health performance. The difference between research findings might be attributed to the different races of study populations, sample sizes, research designs, doses of metal exposure, and methods of metal testing. Furthermore, we found that the negative association with eGFR in the multi-metal model remained significant only for As. The nonsignificant results in multi-metal models for others metals/metalloids might be due to the possible interactions between metals/metalloids and genetic polymorphisms.

Strontium might be beneficial for kidney function, regardless of multi-metal interactions. Participants with the highest urine Sr had a higher eGFR and better performance in kidney function. A cross-sectional study conducted in Hunan Province, China, also found a possible protective effect of urinary Sr on renal function^53^. Nevertheless, limited epidemiological evidence on the relationship between Sr and kidney function has led to inconsistent conclusions. A cohort study focused on serum metals reported that Sr was associated with a decline in annual eGFR and an increased risk of impaired kidney function^40^. Moreover, the biological role of Sr in kidney health is still under researched. Recently, Min et al.^54^ reported that strontium ions protected hearts against myocardial ischemia/reperfusion injury. They found that the expression of endothelial nitric oxide synthase (eNOS) in human umbilical vein endothelial cells (HUVECs) was increased after cultivation with Sr ions under normal conditions. qRT‒PCR analysis also showed that Sr ions stimulated HUVECs to express higher levels of the eNOS gene^54^. A potential mechanism could be that upregulated eNOS resulted in more nitric oxide (NO) synthesis in the glomeruli. NO dilated blood vessels and reduced internal glomerular pressure, resulting in a delay in the decline in eGFR. However, further biological experiments and prospective population studies are needed to verify our findings.

Metals/metalloids could be correlated with each other. After entering into human body in several common ways^10,11^, metals/metalloids may go through similar procedure, such as absorption, distribution, metabolism, and transformation, until they exert biological effects and finally eliminated by organism^55^. Also, the absorption of metals/metalloids at receptor sites may affect the access of other metals or metalloids^8,9,15^. It has been reported that metals/metalloids may interact with each other by more than one pathway^55,56^. It is consistent with our experimental results (Supplemental Table S1) that most metals/metalloids concentrations are correlated in vivo.

In our analysis, mixed metals/metalloids were found to pose a potential threat to kidney health, possibly accompanied by decreased kidney function, an increased risk of kidney damage, and an increased incidence of CKD. A cohort study explored the cause-effects association between co-exposure to 23 kinds of plasma metals/metalloids and decreased kidney function in the Chinese population^40^. Apart from the mentioned cohort, multiple cross-sectional studies reported results consistent with ours, but these previous studies examined fewer metals/metalloids^57,58^. Analysis based on NHANES data found that the mixture of blood Co, Cr, Hg, and Pb was related to the poor performance of kidney function in a dose‒response manner^58^. The mixed As, Cd, V, Co, and Tl in urine was also found to have a dose‒response association with the risk of CKD in another city of China, despite being based on older diabetes mellitus patients^57^. However, studies on the mixed association between metals/metalloids and kidney health are still limited, and few have included enough metals/metalloids. Since metals/metalloids and toxic chemicals are everywhere in actual life, compared to single relations, the health effects of being co-exposed to multiple metals/metalloids are worth considering. Population-based studies with more metals/metalloids are needed for further evidence on this topic. Moreover, the possible mechanism of renal damage caused by mixed metals/metalloids also needs to be clarified in the future.

The association between metals/metalloids and albuminuria was similar to that between metals/metalloids and CKD. In our analysis, CKD was defined as decreased kidney function indicated by a low eGFR or kidney damage marked by albuminuria. Among 138 participants who were diagnosed with CKD, 129 had kidney damage and 31 had decreased kidney function. The possible explanation of the similarity might be that participants who had albuminuria accounted for the majority of individuals with CKD. Future studies with more individuals are needed to expand the population base and validate the results of this study.

Our research has some strengths. First, we had an adequate sample size, which assures the stability and power of our findings. Second, to the best of our knowledge, we measured 21 kinds of urine metals/metalloids simultaneously, which was an absolute advantage compared to other studies on the same topic. Compared to previous research focused on a limited number of metals/metalloids, we can provide more comprehensive evidence for the relationship between metals/metalloids and kidney function. Moreover, our sample was recruited from patients undergoing routine health examinations, which is more representative of the daily urine exposure of the general population in this area.

Limitations were inevitable in our research. First, a cross-sectional design would not be able to explore the cause and outcome relation between metal exposure and damaged kidney function. Second, although our participants were recruited from a well-known hospital in the Chinese middle area, selection bias could not be avoided, and the conclusion was restrained for a certain population. Third, we only collected single morning-time urine and blood samples, which may not be able to reflect long-term metal exposure or kidney function status. Last but not least, as we all live in a complicated natural and social environment, there are far more than 21 kinds of metals/metalloids that could be related to human health, and we were unable to thoroughly examine all options.

## Conclusion

For healthy and non-healthy people, daily exposure to relatively high levels of urinary Cu, Fe, and Zn might be related to increased risks of CKD and albuminuria. Daily exposure to higher urinary Sr might be beneficial for kidney function, while exposure to several other kinds of metals/metalloids might be harmful. Moreover, a mixture of metals/metalloids (predominately Cu) was positively associated with the risks of CKD and albuminuria, and the reduction in eGFR in the mixture was primarily attributed to As. Further longitudinal studies are expected to validate our results.

### Supplementary Information

Supplementary Information.
