# Supplementary material for: The association between urinary metals/metalloids and chronic kidney disease among general adults in Wuhan, China
Source: Sci Rep. 2023 Sep 15;13:15321. doi: 10.1038/s41598-023-42282-z (PMC10504376; doi:10.1038/s41598-023-42282-z)
Supplement: Supplementary file 1 — Supplementary Information. [file 41598_2023_42282_MOESM1_ESM.docx]

**Supplemental Data**

Supplemental Table S1 Spearman correlations between 21 metals

Bold effects represented the non-significantly correlation between metals.

Supplemented Table S2 Associations between single metals and risk of CKD

| Metals | Odds ratio (95% CI) by quartile of metals | | | | *P-trend* |
| --- | --- | --- | --- | --- | --- |
|  | Q1 | Q2 | Q3 | Q4 |  |
| Al | Ref. | 2.11(1.23,3,60) | 2.12(1.25,3,62) | 1.42(0.84,2.42) | 0.17 |
| V | Ref. | 1.12(0.67,1.88) | 1.05(0.61,1.80) | 0.85(0.52,1.39) | 0.53 |
| Cr | Ref. | 1.56(0.93,2.61) | 1.18(0.67,2.07) | 1.32(0.81,2.15) | 0.38 |
| Mn | Ref. | 0.97(0.57,1.65) | 0.97(0.55,1.71) | 1.20(0.74,1.95) | 0.47 |
| **Fe** | Ref. | **2.15(1.25,3.68)** | **1.38(0.75,2.56)** | **2.39(1.42,4,02)** | **<0.05** |
| Co | Ref. | 0.96(0.58,1.59) | 0.99(0.57,1.75) | 0.87(0.52,1.46) | 0.67 |
| Ni | Ref. | 1.01(0.61,1.68) | 0.97(0.57,1.64) | 1.08(0.65,1.80) | 0.82 |
| **Cu** | Ref. | **2.35(1.33,4.16)** | **2.11(1.16,3.85)** | **2.94(1.70,5.11)** | **<0.05** |
| **Zn** | Ref. | **1.69(0.91,3.13)** | **2.31(1.28,4.18)** | **2.35(1.31,4.24)** | **<0.05** |
| As | Ref. | 1.09(0.66,1.80) | 0.97(0.58,1.64) | 0.88(0.51,1.50) | 0.56 |
| Se | Ref. | 0.83(0.50,1.36) | 0.73(0.41,1.28) | 1.09(0.66,1.81) | 0.85 |
| Rb | Ref. | 1.45(0.88,2.38) | 1.07(0.61,1.87) | 1.10(0.64,1.91) | 0.99 |
| Sr | Ref. | 0.93(0.56,1.54) | 0.68(0.38,1.21) | 0.88(0.53,1.44) | 0.44 |
| Ag | Ref. | 1.97(1.17,3.32) | 1.72(0.98,3.02) | 1.31(0.78,2.20) | 0.40 |
| Cd | Ref. | 1.18(0.69,2.03) | 1.07(0.61,1.87) | 1.21(0.70,2.08) | 0.58 |
| Cs | Ref. | 0.71(0.40,1.23) | 1.67(1.03,2.70) | 0.70(0.41,1.21) | 0.82 |
| Ba | Ref. | 0.73(0.43,1.25) | 1.16(0.69,1.96) | 0.81(0.49,1.34) | 0.67 |
| Hg | Ref. | 1.3490.79,2.26) | 1.10(0.63,1.92) | 1.26(0.76,2.09) | 0.53 |
| Tl | Ref. | 0.91(0.52,1.62) | 1.77(1.09,2.88) | 0.95(0.55,1.64) | 0.50 |
| Pb | Ref. | 1.55(0.91,2.62) | 1.52(0.87,2.65) | 1.50(0.91,2.49) | 0.13 |
| U | Ref. | 1.47(0.90,2.39) | 1.13(0.65,1.97) | 0.83(0.49,1.38) | 0.40 |

Models adjusted for age, gender, BMI, marital status, education, family income, smoking, alcohol, hypertension, and diabetes.

Supplemented Table S3 Associations between single metals and risk of Albuminuria

| Metals | Odds ratio (95% CI) by quartile of metals | | | | *P-trend* |
| --- | --- | --- | --- | --- | --- |
|  | Q1 | Q2 | Q3 | Q4 |  |
| Al | Ref. | 2.27(1.29,3.97) | 2,32(1.33,4.03) | 1.58(0.91,2.73) | 0.10 |
| V | Ref. | 1.20(0.71,2.03) | 1.06(0.60,1.85) | 0.89(0.54,1.48) | 0.62 |
| Cr | Ref. | 1.31(0.77,2.24) | 1.03(0.57,1.85) | 1.27(0.78,2.07) | 0.46 |
| Mn | Ref. | 0.94(0.53,1.64) | 1.08(0.77,2.06) | 1.26(0.77,2.06) | 0.32 |
| **Fe** | Ref. | **2.12(1.21,3.73)** | **1.45(0.77,2.74)** | **2.52(1.48,4.31)** | **<0.05** |
| Co | Ref. | 0.88(0.52,1.49) | 0.99(0.56,1.76) | 0.86(0.50,1.46) | 0.65 |
| Ni | Ref. | 1,11(0.66,1.89) | 1.07(0.62,1.83) | 1.16(0.68,1.96) | 0.63 |
| **Cu** | Ref. | **2.23(1.23,4.04)** | **2.20(1.19,4.07)** | **3.03(1.72,5.33)** | **<0.05** |
| **Zn** | Ref. | **1.56(0.84,2.90)** | **2.14(1.18,3.88)** | **2.07(1.15,3.75)** | **0.01** |
| As | Ref. | 1.12(0.67,1.87) | 0.96(0.56,1.66) | 0.89(0.52,1.56) | 0.61 |
| Se | Ref. | 0.75(0.44,1.25) | 0.71(0.40,1.26) | 1.04(0.62,1.75) | 0.92 |
| Rb | Ref. | 1.32(0.79,2.12) | 1.11(0.63,1.94) | 1.06(0.60,1.85) | 0.99 |
| Sr | Ref. | 0.92(0.55,1.55) | 0.64(0.35,1.16) | 0.89(0.54,1.48) | 0.48 |
| Ag | Ref. | 2.34(1.35,4.03) | 1.91(1.06,3.43) | 1.52(0.88,2.61) | 0.24 |
| Cd | Ref. | 1.25(0.73,2.16) | 1.06(0.60,1.86) | 1.06(0.61,1.86) | 0.99 |
| Cs | Ref. | 0.85(0.48,1.50) | 1.91(1.16,3.15) | 0.79(0.45,1.39) | 0.84 |
| Ba | Ref. | 0.77(0.44,1.33) | 1.21(0.71,2.07) | 0.83(0.50,1.39) | 0.76 |
| Hg | Ref. | 1.41(0.81,2.45) | 1.27(0.71,2.24) | 1.40(0.82,2,38) | 0.29 |
| Tl | Ref. | 0.85(0.47,1.53) | 1.65(1.01,2.71) | 0.91(0.53,1.59) | 0.63 |
| Pb | Ref. | 1.39(0.81,2.41) | 1.52(0.86,2.68) | 1.50(0.90,2.51) | 0.12 |
| U | Ref. | 1.43(0.87,2.35) | 1.09(0.61,1.93) | 0.84(0.50,1.42) | 0.42 |

Models adjusted for age, gender, BMI, marital status, education, family income, smoking, alcohol, hypertension, and diabetes.

Supplemented table S4 Associations between single metals and eGFR

| Metals | Relative change (95% CI) by quartile of metals | | | | *P-trend* |
| --- | --- | --- | --- | --- | --- |
|  | Q1 | Q2 | Q3 | Q4 |  |
| Al | Ref. | -0.03(-1.72,1.66) | -0.48(-2.18,1.21) | -0.65(-2.34,1.05) | 0.42 |
| V | Ref. | 0.09(-1.60,1.78) | 1.72(0.03,3.41) | 0.91(-0.78,2.61) | 0.12 |
| Cr | Ref. | 0.62(-1.06,2.31) | 0.02(-1.68,1.72) | -1.13(-2.84,0.57) | 0.16 |
| **Mn** | Ref. | -0.48(-2.17,1.21) | **-1.50(-3.20,0.20)** | **-2.28(-3.97, -0.58)** | **<0.05** |
| Fe | Ref. | 1.97(0.28,3.66) | -0.81(-2.51,0.89) | -0.17(-1.86,1.52) | 0.22 |
| Co | Ref. | -0.86(-2.55,0.83) | -1.98(-3.69, -0.27) | -0.55(-2.31,1.21) | 0.31 |
| **Ni** | Ref. | -0.41(-2.10,1.28) | -0.53(-2.22,1.16) | **-2.29(-3.98, -0.60)** | **0.01** |
| **Cu** | Ref. | -0.52(-2.20,1.16) | -1.60(-3.29,0.09) | **-2.65(-4.33, -0.96)** | **<0.05** |
| **Zn** | Ref. | -1.82(-3.52, -0.12) | **-1.42(-3.14,0.30)** | **-3.33(-5.09, -1.58)** | **<0.05** |
| As | Ref. | -1.27(-2.96,0.42) | **-2.63(-4.32, -0.93)** | **-2.73(-4.42, -1.03)** | **<0.05** |
| Se | Ref. | -0.77(-2.47,0.93) | **-1.82(-3.51, -0.12)** | **-2.58(-4.28, -0.87)** | **<0.05** |
| Rb | Ref. | -0.43(-2.14,1.27) | -1.17(-2.87, 0.53) | -0.43(-2.14,1.27) | 0.60 |
| Sr | Ref. | 1.01(-0.68,2.69) | **1.86(0.17,3.55)** | **2.09(0.39,3.78)** | **0.01** |
| Ag | Ref. | 1.09(-0.60,2.78) | 2.15(0.45,3.84) | 1.54(-0.16,3.24) | 0.05 |
| Cd | Ref. | -1.69(-3.38, -0.01) | -1.43(-3.14,0.29) | **-2.85(-4.61, -1.09)** | **<0.05** |
| Cs | Ref. | -1.26(-2.95,0.44) | **-1.77(-3.47, -0.08)** | **-1.86(-3.56, -0.16)** | **0.03** |
| Ba | Ref. | 2.03(0.35,3.70) | 1.89(0.20,3.58) | 1.38(-0.31,3.07) | 0.14 |
| Hg | Ref. | -1.25(-2.94,0.44) | -0.26(-1.96,1.44) | -0.51(-2.21,1.18) | 0.80 |
| Tl | Ref. | -1.19(-2.88,0.50) | 0.43(-1.27,2.13) | -0.06(-1.76,1.63) | 0.67 |
| Pb | Ref. | 1.50(-0.19,3.19) | 1.65(-0.04,3.35) | 0.63(-1.07,2.33) | 0.48 |
| U | Ref. | -1.18(-2.87,0.52) | **-1.74(-3.43, -0.42)** | **-2.11(-3.80, -0.42)** | **0.01** |

Models adjusted for age, gender, BMI, marital status, education, family income, smoking, alcohol, hypertension, and diabetes.
